# Identification of collateral sensitivity and evolutionary landscape of chemotherapy-induced drug resistance using cellular barcoding technology

**DOI:** 10.3389/fphar.2023.1178489

**Published:** 2023-07-11

**Authors:** Nurseda Danisik, Kubra Celikbas Yilmaz, Ahmet Acar

**Affiliations:** Department of Biological Sciences, Middle East Technical University, Universiteler Mah, Ankara, Türkiye

**Keywords:** collateral sensitivity, drug resistance, cellular barcoding, colorectal, cancer

## Abstract

**Background:** One of the most significant challenges impeding cancer treatment effectiveness is drug resistance. Combining evolutionary understanding with drug resistance can pave the way for the identification of second-line drug options that can overcome drug resistance. Although capecitabine and irinotecan are commonly used therapeutic agents in the treatment of CRC patients, resistance to these agents is common. The underlying clonal dynamics of resistance to these agents using high-resolution barcode technology and identification of effective second-line drugs in this context remain unclear.

**Methods and materials:** Caco-2 and HT-29 cell lines were barcoded, and then capecitabine and irinotecan resistant derivatives of these cell lines were established. The frequencies of barcodes from resistant cell lines and harvested medium, longitudinally, were determined. Collateral drug sensitivity testing was carried out on resistant Caco-2 and HT-29 cell lines using single agents or drug combinations. The SyngeryFinder tool was used to analyse drug combination testing.

**Results:** In Caco-2 and HT-29 cell lines, barcode frequency measurements revealed clonal dynamics of capecitabine and irinotecan formed by both pre-existing and *de novo* barcodes, indicating the presence of polyclonal drug resistance. The temporal dynamics of clonal evolution in Caco-2 and HT-29 cell lines were demonstrated by longitudinal analysis of pre-existing and *de novo* barcodes from harvested medium. In Caco-2 and HT-29 cell lines, collateral drug sensitivity revealed a number of drugs that were effective alone and in combination.

**Conclusion:** The use of barcoding technology reveals the clonal dynamics of chemotherapy-induced drug resistance not only from harvested cell populations, but also from longitudinal sampling throughout the course of clonal evolution. Second-line drugs that sensitize drug-resistant CRC cell lines are identified through collateral drug testing.

## Background

Cancer is a global health issue that can affect people of all ages and genders, and it is responsible for the world’s second-highest disease-related death rate (Sung et al., 2021). Colorectal cancer (CRC) is one of the five most common cancer types (breast, lung, colon-rectum, prostate, and stomach) and the second most deadly cancer type (9.4%) (Sung et al., 2021). The intratumour heterogeneity (ITH) of CRC has aided in its classification based on genomic and transcriptomic profiling (Chowdhury et al., 2021). CRC genomic profiling has defined two types of cancer: hypermutated cancer, which includes microsatellite instable (MSI), and non-hypermutated cancer, which includes microsatellite stable (MSS) (Muzny et al., 2012). Transcriptomic classification identified four subtypes based on molecular subtyping: CMS1 (MSI-immune), CMS2 (MSS-canonical), CMS3 (MSS-metabolic), and CMS4 (MSS-mesenchymal). ITH is known to fuel drug resistance, so accurate identification of ITH is required for tailoring better treatment modalities (Guinney et al., 2015).

The heterogeneous nature of tumour cells is important in mediating the drug resistance mechanisms (Burrell and Swanton, 2014; Yalcin et al., 2020). Methods for tracking tumour clonal evolution based on identification of tumour subclones have the potential to investigate acquired or *de novo* drug resistance (Hata et al., 2016; Acar et al., 2020; Caravagna et al., 2020). Viral barcoding methods are useful tools in cellular barcoding technology, and they are frequently used to track clonal dynamics and evolution in cellular populations (Bhang et al., 2015; Acar et al., 2020). Cellular barcoding technology, as compared to traditional next-generation sequencing approaches, provides high resolution solutions in a controlled *in vitro* or *in vivo* environment and enables the identification of ongoing clonal evolution (Porter et al., 2014; Acar et al., 2020; Sankaran et al., 2022). Without the power of cellular barcoding technology, for example, it is limited to deconstructing underlying mechanisms of drug resistance in patient samples because it is often impractical for identifying pre-existing or *de novo* drug resistance mechanisms. A few studies, including ours, have recently used this technology to shed light on the complexities of drug resistance by quantifying both pre-existing and *de novo* drug resistance in an experimental evolution platform (Hata et al., 2016; Acar et al., 2020). With the help of barcoding technology, an in-depth understanding of clonal dynamics will enable the true identification of the number of subclones present or emerging as a result of resistance, enabling more effective treatment modalities.

CRC is commonly treated with surgery, radiotherapy, chemotherapy, and targeted therapies (NICE, 2020). Oxaliplatin (OX), irinotecan (IRI), SN-38, 5-fluorouracil (5-FU), and capecitabine (CAPE) are the most commonly used chemotherapeutic agents for CRC patients (Gustavsson et al., 2015). Chemotherapeutic agents disrupt DNA synthesis, resulting in the formation of Double-Strand Breaks (DSB) (Jekimovs et al., 2014). Chemotherapeutic reagents inducing DSB activates DNA damage response (DDR) pathway (Hosoya and Miyagawa, 2014). However, overwhelmed DDR pathway causes imperfect DNA repair, and this may lead to chromosomal instability, deletions, and rearrangements (Ray and Raghavan, 2021). Although chemotherapy is an important part of cancer treatment strategies, the ability to achieve successful results is limited by drug resistance (Housman et al., 2014). Drug resistance is a significant problem in the treatment of cancer, limiting treatment strategies and effective drug options (Vasan et al., 2019). Combination therapies have been widely used as an alternative, but the toxicity that has resulted has paved the way for second-line therapy solutions to be proposed (Hirsch et al., 2016; Mokhtari et al., 2017; Fang et al., 2019). Tumour cells that have developed resistance to one chemotherapeutic agent may be more susceptible to another, a phenomenon known as collateral drug sensitivity (Imamovic and Sommer, 2013; Zhao et al., 2016; Dhawan et al., 2017; Acar et al., 2020). This phenomenon was initially studied in ecological systems where the cost of resistance can come at the expense of a new phenotype, which in the case of cancer is likely to open a window of opportunity for treatment (Merlo et al., 2006; Gatenby et al., 2009). The drugs that will be administered during second-line therapy can be determined by collateral sensitivity to previously administrated chemotherapeutics following acquired resistance to drugs in first-line therapy. This approach has previously been shown to be effective in bacteria (Imamovic and Sommer, 2013; Nichol et al., 2015; Pál et al., 2015), malaria (Kirkman et al., 2018), and cancer (Zhao et al., 2016; Wang et al., 2018; Acar et al., 2020), but the latter still needs to be extensively studied.

Here, using cellular barcoding technology, we were able to identify the clonal dynamics of the drug resistance to capecitabine and irinotecan in the Caco-2 and HT-29 cell lines, respectively. Through using barcode frequency measurements, it was possible to detect both pre-existing and *de novo* barcodes in drug resistant derivatives of Caco-2 and HT-29 cell lines which indicated polyclonal drug resistance. Also, we demonstrated temporal evolution of barcodes from monthly harvested media, enabling us to monitor drug resistance in a non-destructive way. In addition, we found that SN-38 and cetuximab could operate as collateral sensitive drugs in capecitabine-resistant Caco-2 cells, supported further by their additive interactions with capecitabine. The combination of capecitabine and irinotecan induced strong collateral sensitivity with an additive effect, whereas capecitabine alone was unable to promote collateral sensitivity in irinotecan-resistant HT-29 cells. Moreover, dabrafenib by itself, in contrast, was sufficient to induce collateral sensitivity in irinotecan-resistant HT-29 cells. Together, we have identified collaterally sensitive drugs in Caco-2 and HT-29 cell lines while also determining clonal dynamics of drug resistance under capecitabine and irinotecan treatment.

## Methods and materials

### Cell culture

Caco-2 cell line was cultured in MEM-Eagle Earle’s Salts medium (Biological Industries, Israel) supplemented with 20% (v/v) Fetal Bovine Serum (FBS) (Biological Industries, Israel), 1% (v/v) Penicilin-Sreptomycin (Biological Industries, Israel), 1% (v/v) L-Glutamine (Biological Industries, Israel), 1% (v/v) non-essential amino acids (Biological Industries, Israel). HT-29 cell line was maintained in DMEM High Glucose Medium (Biological Industries, Israel) supplemented with 1% (v/v) Penicilin-Sreptomycin (Biological Industries, Israel) and 1% (v/v) L-Glutamine (Biological Industries, Israel). PCR-based method was used to confirm *mycoplasma* negativity in the cell lines.

### Cellular barcoding of the cell lines

The CloneTracker™ lentiviral barcode library (Cellecta, United States) was used for the barcoding of Caco-2 and HT-29 cell lines. Lentiviruses were generated in HEK293T cell line using barcode library, pCMV-VSVG (Addgene: 8,454), and Delta 874. LV (Addgene:8,455). Lentiviral infection was performed using 0.8 μg/mL polybrene. Multiplicity of infection (M.O.I.) 0.1 was achieved during lentiviral titration experiments and puromycin concentrations of 12.5 μg/mL for Caco-2 and 1.5 μg/mL HT-29 cell lines were used.

### Generation of capecitabine-resistant Caco-2 and irinotecan-resistant HT-29 cell lines

A previously frozen cryovial of 4 × 10^6^ barcoded Caco-2 and HT-29 cell were thawed and seeded into 15 cm cell culture dishes. When the cells were 70%–80% confluent, 2 × 10^6^ cells per dish were seeded into four different 15 cm dishes with 25 mL medium (DMSO Control, Replica A, B, C) and 2 × 10^6^ cell pellets were stocked as initial cell population. For Caco-2 cell line, mediums in the dishes were changed twice a week with fresh mediums containing IC50 dose (4 months) and subsequently 2x IC50 dose (2 months) of capecitabine, for HT-29 cell line IC50 dose (6 months) of irinotecan. Caco-2 and HT-29 cell lines treated with DMSO were given the same amount of DMSO used in dissolving compounds as fresh medium.

### Barcode detection using next-generation sequencing

Following the end points of 6 months for each cell line, harvested cell lines were faced to genomic DNA isolation using GeneJET Genomic DNA isolation kit (ThermoFisher, United States). Similarly, collected medium was centrifuged at 1,200 rpm and the collected pellet was used for genomic DNA isolation. For library preparation, barcode amplicons were amplified according to manufacturer’s guidelines provided by the Cellecta. Barcode sequences composed of 14 bp and 30 bp length variable nucleotides were sequenced on MiSeq Platform (Illumina, Inc.) using 150 bp pair-end method.

### The bioinformatics analysis of barcodes

The Illumina MiSeq Platform provided the barcode sequencing results of drug resistant harvested cell populations, their initial and DMSO controls and medium samples FASTQ format. FASTQC was used to evaluate the reads, and those with Phred scores less than 20 were excluded from further investigation. Trimmomatic was used to trim the FASTQ reads (parameter MINLEN:147), and pair-end reads less than 147 bp were eliminated (Bolger et al., 2014). Trimmomatic (parameters HEADCROP:20 CROP:48 for forward reads, HEADCROP:79 CROP:48 for reverse reads) was used to detect variable unique barcodes after trimming the constant nucleotides at both ends (20 bp and 79 bp) (48 bp). According to the Cellecta barcode library excel file (Cellecta-NGS-QC-CloneTracker-XP-10x1MBarcode3-Lib-RFP.xlsx), one million barcode sequences were re-generated computationally. Using the R package “insect,” the regenerated sequences were saved as a FASTA file. Detected barcode sequences were indexed using the Salmon index function in Salmon with the -k 47 parameters (Patro, 2017). The number of barcodes read in forward and reverse directions was counted and written to an SF file using the Salmon function, which was then converted to a JSON file. The built-in library JSON was used to write and read barcode counts in JSON file format.

The detected unique barcode counts were classified based on their frequencies in capecitabine-resistant Caco-2 and irinotecan-resistant HT-29 biological replicates (A, B, C) and their DMSO controls. To determine the phenotypes of the barcodes, the following growth rate formula was used (Acar et al., 2020).

$$r=\frac{1}{T}\log\left(\frac{f_{R}}{f_{0}}\right)$$

Here, f_R_ represents the frequency of the barcode(s) in the replicate, f_0_ represents the maximum frequency of all barcodes in the corresponding DMSO control, and T represents the time (week) between the first drug treatment and the end of the experiment. Barcode counts had less than 2 in all resistant samples and DMSO control were excluded from the analysis. Frequency of all barcodes in the corresponding DMSO control was used as the f_0_ value to calculate the growth rate of the barcodes. According to the calculated growth rates, the barcodes have a positive growth rate, and those detected at least two replicates were classified as “pre-existing”, the barcodes have a positive growth rate, and those detected just in one replicate were classified as “*de novo*”; the remaining detected barcodes were classified as “sensitive”, which had a negative growth rate.

### Harvesting used medium for temporal tracking of barcodes

The used medium was changed with fresh medium including drugs twice a week during the establishment of capecitabine-resistant Caco-2 and irinotecan-resistant HT-29 cell lines, and the floating dead cells in the used mediums were collected as pellets with each medium change. The genomic DNA from these cell pellets at five equal intervals (once every 4 weeks) was isolated and barcode sequencing were performed to track the frequency changes of the resistant barcodes in the cell populations.

### Dose response curve analysis

Capecitabine-resistant Caco-2 and irinotecan-resistant HT-29 ells were seeded into 96 well plates (10 × 10^3^ cells/well), along with their DMSO control and initial cells. Cells were treated with drugs after 24 h. The following drugs were used: capecitabine (LC Laboratories, United States), irinotecan (Adooq, United States), SN-38 (Adooq, United States), and Cetuximab (Adooq, United States). The MTT cell viability assay was performed after 72 h of drug treatment.

### Synergy testing

Capecitabine-resistant Caco-2 cells and irinotecan-resistant HT-29 cells, as well as their DMSO controls, were seeded into 96-well plates (10 × 10^3^ cells/well). After 24 h, capecitabine-resistant Caco-2 and its control cells were given SN-38/capecitabine and cetuximab/capecitabine drug combinations, while HT-29 and its control cells were given dabrafenib/irinotecan and capecitabine/irinotecan drug combinations. Capecitabine and irinotecan were used as background drugs in drug combination assays, and their doses remained stable as 2x IC50 and 1XIC50, respectively. Capecitabine-resistant and control Caco-2 cells were given four doses of SN-38 and cetuximab in combination with capecitabine; irinotecan-resistant and control HT-29 cells were treated with five doses of dabrafenib and capecitabine. The MTT cell viability assay was performed after 72 h of incubation. Cell viability based on dose response curves was calculated using control wells, and synergy scores of drug combinations using cell viability results were determined using SynergyFinder 2.0 software (University of Helsinki, Finland). Using this software, the scores between −10 and +10 showed additive effect, +10 and more as synergistic, and −10 and less as antagonistic.

### Growth rate analysis

10 × 10^3^ cells per well capecitabine-resistant Caco-2, irinotecan-resistant HT-29 cells, DMSO controls, and initial cell populations were seeded into three separate 96 well plates. After 24 h, DMSO control Caco-2 and HT-29 cells and capecitabine-resistant Caco-2 and irinotecan-resistant HT-29 cells were treated with DMSO, capecitabine, and irinotecan respectively, while full fresh growth medium was added onto the initial control cells. After 24, 48, and 72 h of incubation time in the incubator, absorbance values were measured using a microplate spectrophotometer (Multiskan GO; Thermo Fisher Scientific, United States) at 570 nm wavelength. GraphPad Prism 8 was used to calculate cell proliferation rates based on absorbance values (GraphPad Software Inc., United States).

### Scratch assay

Migration of drug resistant, DMSO control and drug resistant Caco-2 and HT-29 cell lines were examined using scratch assay. Equal number of cells per cell line groups (3.5 × 10^5^/well for Caco-2 and 4 × 10^5^/well for HT-29) were seeded in a 24-well plate containing growth. After cells reached to approximately 80% of confluency, cells were treated with 2 μg/mL Mitomycin (Serva, VWR International) for 2 h. Scratches were made using 200 μL pipette tip and cell were washed three times to remove the debris. During scratch assay, DMSO control Caco-2 and HT-29 cells were treated with DMSO and capecitabine-resistant Caco-2 and irinotecan-were treated with capecitabine and irinotecan respectively, while full fresh growth medium were added onto the initial control cells. Migration of cells was monitored every 4 hours where the images were taken using Nikon Eclipse Ti2e microscope. The area of closure was calculated using previously published ImageJ software plugin (Suarez-Arnedo et al., 2020).

### RNA extraction

Total RNA was isolated and purified by using the Invitrogen PureLink RNA Mini Kit (Thermo Fisher Scientific, United States) according to the manufacturer’s instructions. Total RNA was isolated from frozen 2 × 10^6^ cell pellets of Caco-2 DMSO control, Capecitabine-resistant Caco-2 Replicates A, B and C; HT-29 DMSO control, Irinotecan-resistant HT-29 Replicates A, B and C. Quality and concentration of the isolated RNA samples were controlled by using spectrophotometer (Biodrop, United Kingdom).

### cDNA generation

Reverse transcription protocol was applied on 1 µg total RNA of each sample. The RNA samples were diluted with d T)_23_ VN (50 µM) (2 µL), ProtoScript II Reaction Mix 2X (10 µL), ProtoScript II Enzyme Mix 10 X (2 µL) and Nuclease-free water (to were added to complete the reaction solution to 20 µL (ProtoScript II First Strand cDNA Synthesis Kit, New England BioLabs, United States).

### Real-Time PCR

Real-time PCR experiments were conducted by using CFX Real-Time PCR Systems (BioRad, United States). 5 μL samples of cDNAs in 15 µL PCR buffer GoTaq q PCR Master Mix (Promega, United States). The following primers were used:

ABCB1-Forward: ACA​GAA​AGC​GAA​GCA​GTG​GT.

ABCB1-Reverse: ATG​GTG​GTC​CGA​CCT​TTT​C.

Tm: 60°C

GAPDH-Forward: TGG​TCA​CCA​GGG​CTG​CTT​TT.

GAPDH-Reverse:ACTCCACGACGTACTCAGCG.

Tm: 60°C

### Statistical analysis

Every experiment had at least three biological replicates, as well as at least three technical replicates. GraphPad Prism 8 was used for dose response curve data analysis (GraphPad Software Inc., United States). The statistical significance was determined using the Student’s t-test, two-way Anova, and nonlinear regression. *p*-values less than 0.05 were considered statistically significant. All results were given as mean SEM.

## Results

### Establishment of barcoded capecitabine and irinotecan resistant Caco-2 and HT-29 cell lines, respectively

**FIGURE 1:**
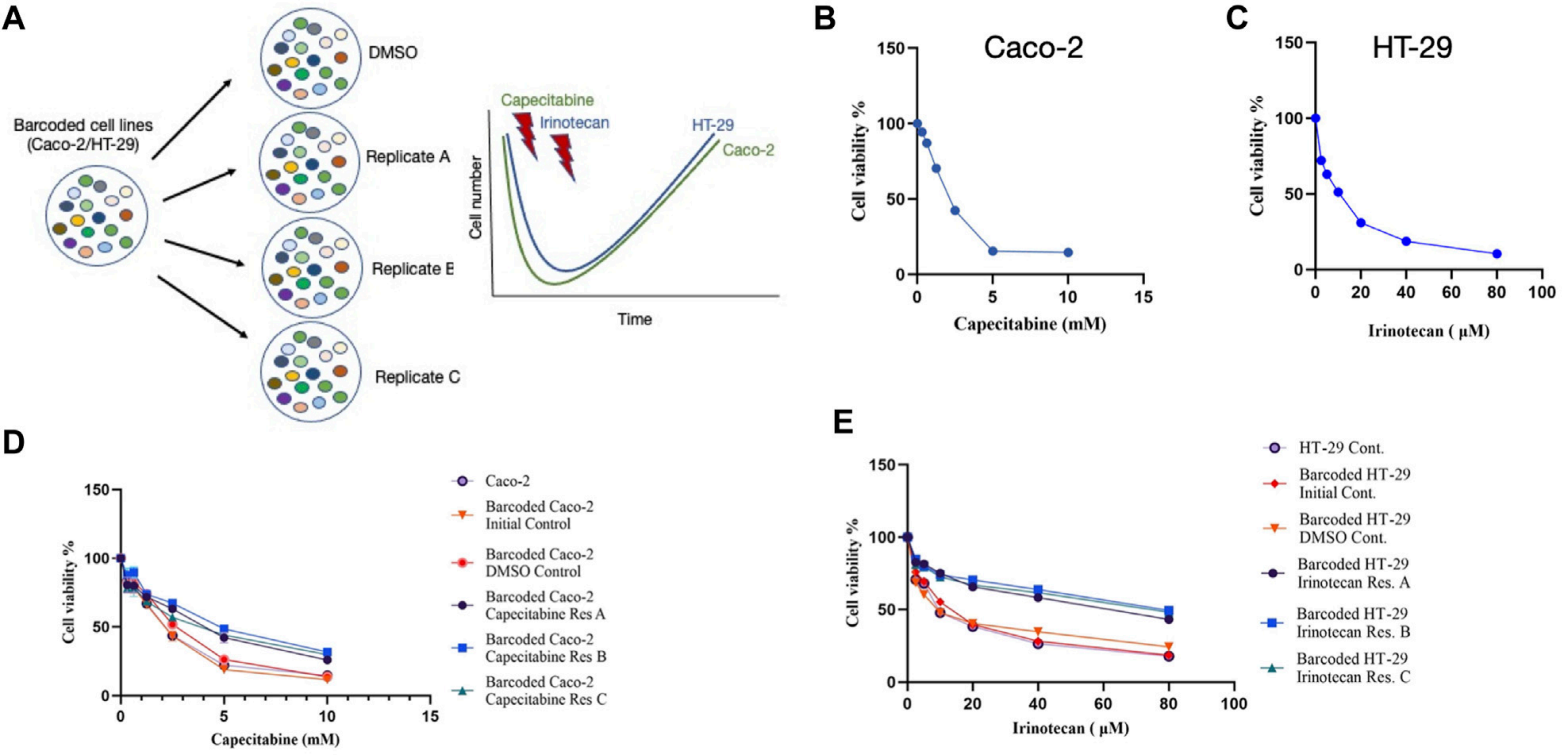
Experimental design and establishment of drug resistant cell lines. **(A)**. Schematic description of the experimental set-up for barcoding of Caco-2 and HT-29 cell lines alongside with drug treatment schedules. **(B)**. MTT cell viability assay results for capecitabine treatment in Caco-2 cell line. **(C)**. Dose response curve analysis of irinotecan treatment in HT-29 cell line based on MTT cell viability assay. **(D)**. MTT cell viability results of dose response analysis for capecitabine-resistant lines and **(E)**. irinotecan-resistant lines *versus* barcoded DMSO, initial and non-barcoded parental cell lines. Error bars represent SEM.

The cellular barcoding method was used to investigate the clonal dynamics in Caco-2 and HT-29 cell lines under capecitabine and irinotecan selection, respectively (Figure 1A). To ensure low lentiviral infection efficiency of barcode library into these cell lines, multiplicity of infection (M.O.I) efficiency less than 0.1% was targeted. Capecitabine and irinotecan were used to establish their resistant counterparts in barcoded Caco-2 and HT-29 cells, respectively. First, we wanted to determine the half-maximal inhibitory concentrations (IC50) of capecitabine and irinotecan on Caco-2 and HT-29, respectively, using the MTT cell viability assay. The IC50 values of capecitabine and irinotecan were found as 2.12 mM and 8.851 μM on barcoded Caco-2 and HT-29 cell lines, respectively (Figures 1B,C). Following that, we divided barcoded Caco-2 cells into four parallel groups of equal cell numbers, with one replicate treated with DMSO and the remaining three replicates treated with capecitabine (replicate A, B and C). To induce capecitabine resistance in replicate A, B, and C of barcoded Caco-2 cell lines, IC50 concentrations of capecitabine were administered for 4 months, followed by incremental 2XIC50 concentrations of capecitabine for an additional 2 months. Of note, we did not observe capecitabine resistance after 4 months of IC50 concentration of capecitabine exposure on barcoded Caco-2 cells and for this reason we applied 2XIC50 concentration of capecitabine on Caco-2 cell lines. Following 6 months of capecitabine treatment, DMSO and the initial population of barcoded Caco-2 cell lines were subjected to an MTT assay. As a result, we found increased and consistent capecitabine resistance in all three replicates of barcoded Caco-2 cell lines when compared to the DMSO control and the initial Caco-2 cell lines. (Figure 1D). Resistance fold changes in capecitabine resistant barcoded replicates A, B, and C in comparison to DMSO control were 1.4-, 1.87-, and 1.36-fold (Supplementary Table S1), indicating a similar degree of resistance achieved in three parallel drug resistant replicates, with replicate B being the most resistant. After this, we aimed to establish irinotecan resistance in a barcoded HT-29 cell line, as we had done previously. Barcoded HT-29 cells were treated for 6 months with the IC50 concentration of irinotecan (8.851 μM) to establish an irinotecan-resistant HT-29 cell line. Following this, irinotecan resistance in barcoded HT-29 cell lines (replicate A, B and C) were confirmed in relative to barcoded control DMSO and initial HT-29 cell lines using the MTT cell viability assay. This assay exhibited elevated degree of resistance to irinotecan barcoded HT-29 replicates A, B and C relative to control DMSO and initial HT-29 cell lines (Figure 1E). Moreover, quantification of resistance to irinotecan in barcoded HT-29 replicates A, B and C were found as 4.1-, 5.15-, and 4.55-fold increase with respective barcoded control DMSO HT-29 cell line, respectively (Supplementary Table S2). These findings demonstrated successful barcoding of Caco-2 and HT-29 cell lines, as well as the establishment of barcoded capecitabine and irinotecan resistant derivatives, respectively, in three parallel replicates.

### Measurement of clonal selection in capecitabine resistant Caco-2 and irinotecan resistant HT-29 cell lines

Understanding drug resistance mechanisms such as pre-existing or *de novo* within the system is critical. Because the lentiviral barcode library integrated into the Caco-2 and HT-29 cell lines contains consensus forward and reverse primer sequences, it could be possible to calculate changes in individual barcode frequencies after drug treatment in comparison to the control DMSO sample. Furthermore, if the same barcodes were selected in parallel drug resistant cell lines, there is pre-existing drug resistance; whereas barcodes detected with increased frequency in parallel replicates but not shared in any of them will suggest the presence of potentially *de novo* drug resistance (Acar et al., 2020). Based on this, we performed barcode amplicon next-generation sequencing on capecitabine resistant Caco-2 cell lines and irinotecan resistant HT-29 cell lines in the initial, DMSO, and replicates A, B, and C. End point harvested cells were then subjected to genomic DNA isolation and barcode amplicon library preparation prior to sequencing. As before, each barcode was assigned to a phenotype, and the growth rate in each condition was calculated based on the detected frequencies of the barcodes.

**FIGURE 2:**
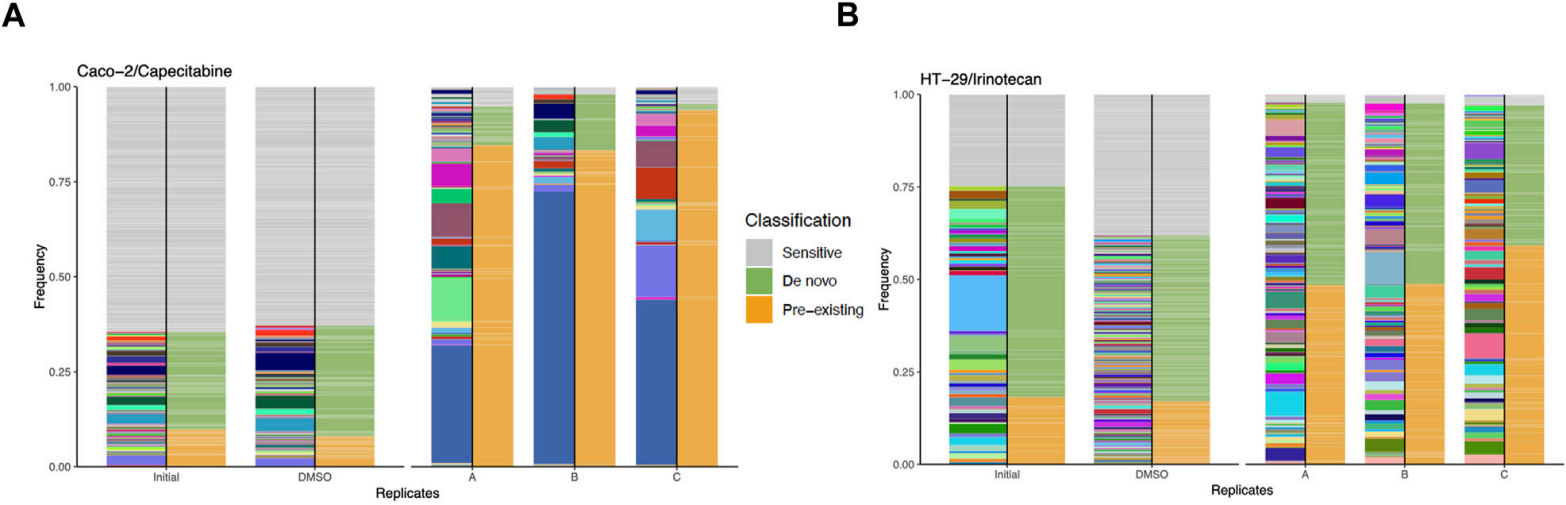
Barcode frequency measurements to assess evolutionary dynamics in drug resistant cells. **(A)**. Frequency measurements of each sample groups, namely, initial, DMSO and capecitabine resistant replicate A, B and C in Caco-2 cells are shown. **(B)**. Frequency distributions of each sample groups are shown with the following samples: HT-29 cell line, initial, DMSO, irinotecan resistant replicate A, B and C. The phenotypes of barcodes are indicated with different colours (amber: pre-existing, olive green: *de novo*, silver grey: sensitive) on the right-hand side of bars. Barcodes with positive growth rate are classified as pre-existing or *de novo* (see “Methods” for the further details about barcode classification).

Bioinformatics analysis of capecitabine resistant Caco-2 cell line barcode sequences revealed 68, 48, and 62 unique pre-existing barcodes in replicates A, B, and C, respectively (Supplementary Table S3). Moreover, in replicates A, B, and C, the number of resistant *de novo* barcodes detected was 83, 35, and 34, respectively (Supplementary Table S3). Furthermore, in barcoded Caco-2 cell line, there were 6,547 barcodes in initial and 463, 361, 580 barcodes left in replicates A, B, C, respectively. In replicates A, B, and C, frequency calculations of pre-existing barcodes (amber colour) provided 84.62%, 83.23%, and 93.83%, respectively. (Figure 2A). Furthermore, in replicates A, B, and C, *de novo* resistant barcode frequencies (olive green colour) were found to be 10.17%, 14.81%, and 1.71%, respectively. (Figure 2A). Finally, sensitive barcode frequencies (silver grey colour) exhibited negative growth rate in capecitabine-treated replicates A, B, and C were identified (Figure 2A). We then looked at irinotecan-resistant HT-29 cell lines in the same way we approached at capecitabine-resistant Caco-2 cells. In replicates A, B, and C of irinotecan-resistant HT-29 cell lines, the unique number of pre-existing barcodes was detected as a number of 52, 57, and 47, respectively. (Supplementary Table S4). Moreover, in replicates A, B, and C of irinotecan-resistant HT-29 cell lines, the number of resistant *de novo* barcodes were detected as 89, 82, and 78, respectively (Supplementary Table S4). Also, in barcoded HT-29 cell line, there were 7,511 barcodes in initial and 1,536, 1,556, 1,565 barcodes left in replicates A, B, C, respectively. Furthermore, in replicates A, B, and C, the frequencies of pre-existing resistant barcodes (amber colour) were found to be 48.44%, 48.74%, and 59.14%, respectively (Figure 2B). Furthermore, within the same groups, *de novo* resistant barcode frequencies (olive green colour) were detected as 49.29%, 48.90%, and 37.88%, respectively (Figure 2B). Finally, within the same replicates, barcode frequencies for sensitive ones (silver grey colour) were identified (Figure 2B). Using barcoding technology, we were able to determine clonal selection under capecitabine resistance in Caco-2 and irinotecan resistance in HT-29 cell lines, where both pre-existing and *de novo* resistance was present in all parallel replicates, indicating the presence of polyclonal drug resistance in our setting.

### Temporal tracking of clonal evolution in capecitabine resistant Caco-2 and irinotecan resistant HT-29 cell lines

**FIGURE 3:**
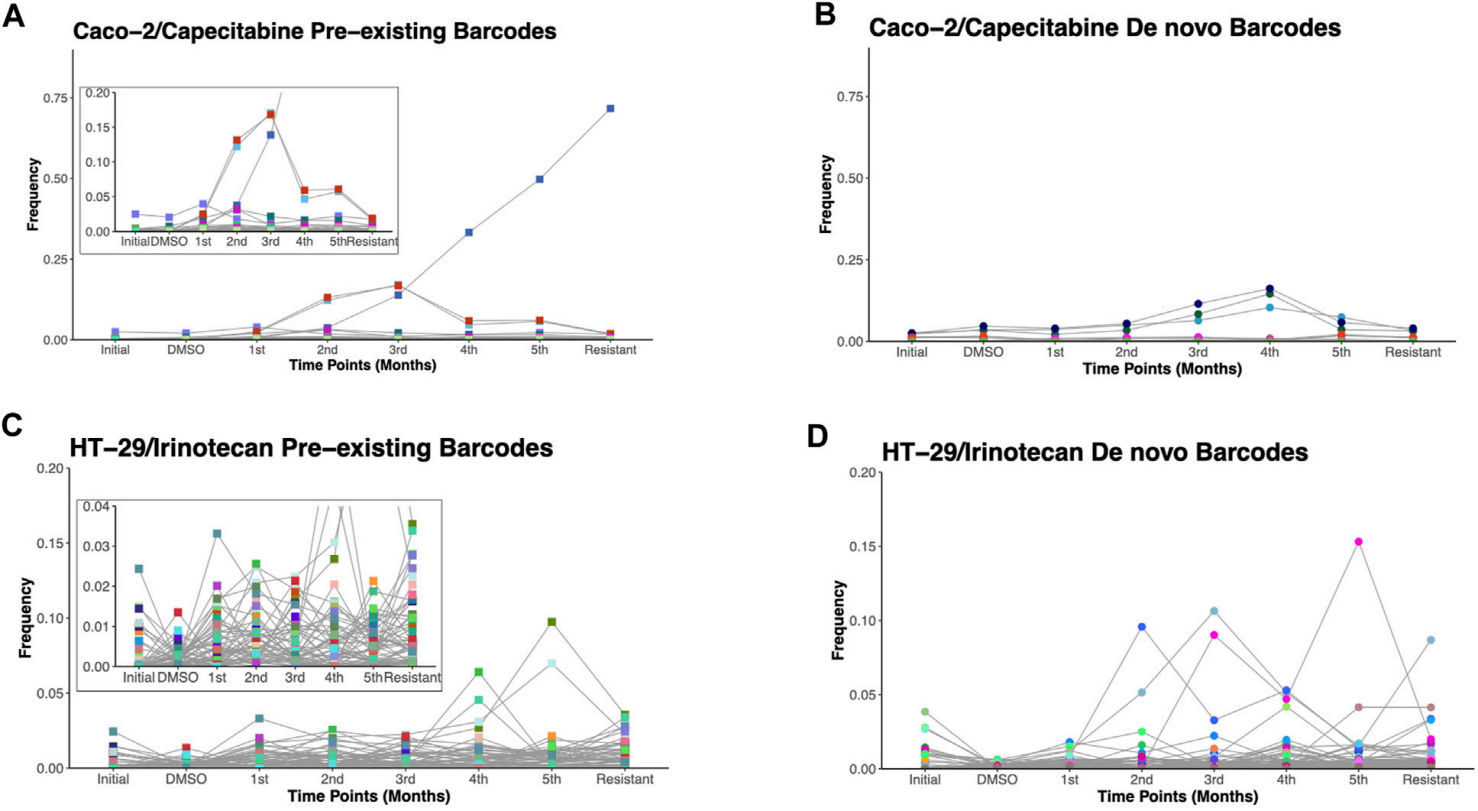
Temporal non-destructive analysis of clonal evolution in drug resistant cells. **(A)**. Pre-existing and **(B)**. de novo floating barcode frequencies of Caco-2 replicate B cell line under capecitabine treatment for duration of 6 months. **(C)**. Pre-existing and **(D)**. de novo floating barcode frequencies of HT-29 replicate B cell line under irinotecan treatment for duration of 6 months. Last data point demonstrating barcode frequency nearly matches the barcode frequency in harvested (resistant) sample point. Distinct colours represent each individual barcodes.

Due to the ability of barcodes to integrate into DNA and dead cells to release their DNA particles into cell growth medium, we sought to track these barcodes for 6 months in capecitabine-treated Caco-2 and irinotecan-treated HT-29 cell lines. Monthly samples collected from used cell growth medium were subjected to gDNA isolation, barcode library preparation, and amplicon sequencing, as previously performed. Barcode sequencing in five different mediums collected at equal intervals for the most capecitabine resistant Caco-2 replicate B identified 47 pre-existing and 33 *de novo* barcodes with time-dependent positive growth frequencies and matching nearly their end points from the harvested population (Figures 3A,B). In capecitabine resistant Caco-2 replicate B, one of the pre-existing barcodes (ID number 82195) were detected with the greatest frequency change over time (Figure 3A). After 4 months, we observed a decrease but still positive growth rate of two barcodes (barcode ID numbers 168122 and 545,550) within the population presumably due to the switch from 1XIC50 to 2XIC50 concentration of capecitabine (Figure 3A). The increase in the concentration of capecitabine might be responsible for affecting the growth rates of these 2 barcodes. Furthermore, even when capecitabine concentrations were increased, remaining barcodes were detected with a positive growth rate in the population at a similar frequency (Figure 3A). We then used the same method to look into the temporal clonal evolution of irinotecan resistance in HT-29 cells. Medium samples were collected from five equal time points of the most irinotecan resistant HT-29 replicate B for this purpose. The detection of 56 pre-existing and 81 *de novo* barcodes with positive growth rate was confirmed by barcode analysis (Figures 3C,D). All of the pre-existing and *de novo* barcodes showed an increase in frequency and were closely matched to the barcode frequencies of the harvested population (Figures 3C,D). For example, the top two highest pre-existing barcode frequencies detected in the HT-29 cell line during irinotecan selection (barcode ID numbers 96965 and 243,910) appeared to dominate the population over a 6-month period. Last, we investigated sensitive barcode frequencies in the harvested medium which exhibited negative growth rate both in capecitabine-resistant Caco-2 replicate B and irinotecan-resistant HT-29 replicate B cell lines over the period of 6 months (Supplementary Figures S1A, B). Thus, polyclonal drug resistance detected in capecitabine resistant Caco-2 and irinotecan resistant HT-29 cell lines was confirmed by temporal detection of increased barcode frequencies in a non-destructive way.

### SN-38 and cetuximab are effective two drugs in inducing collateral sensitivity in capecitabine resistant Caco-2 cells

**FIGURE 4:**
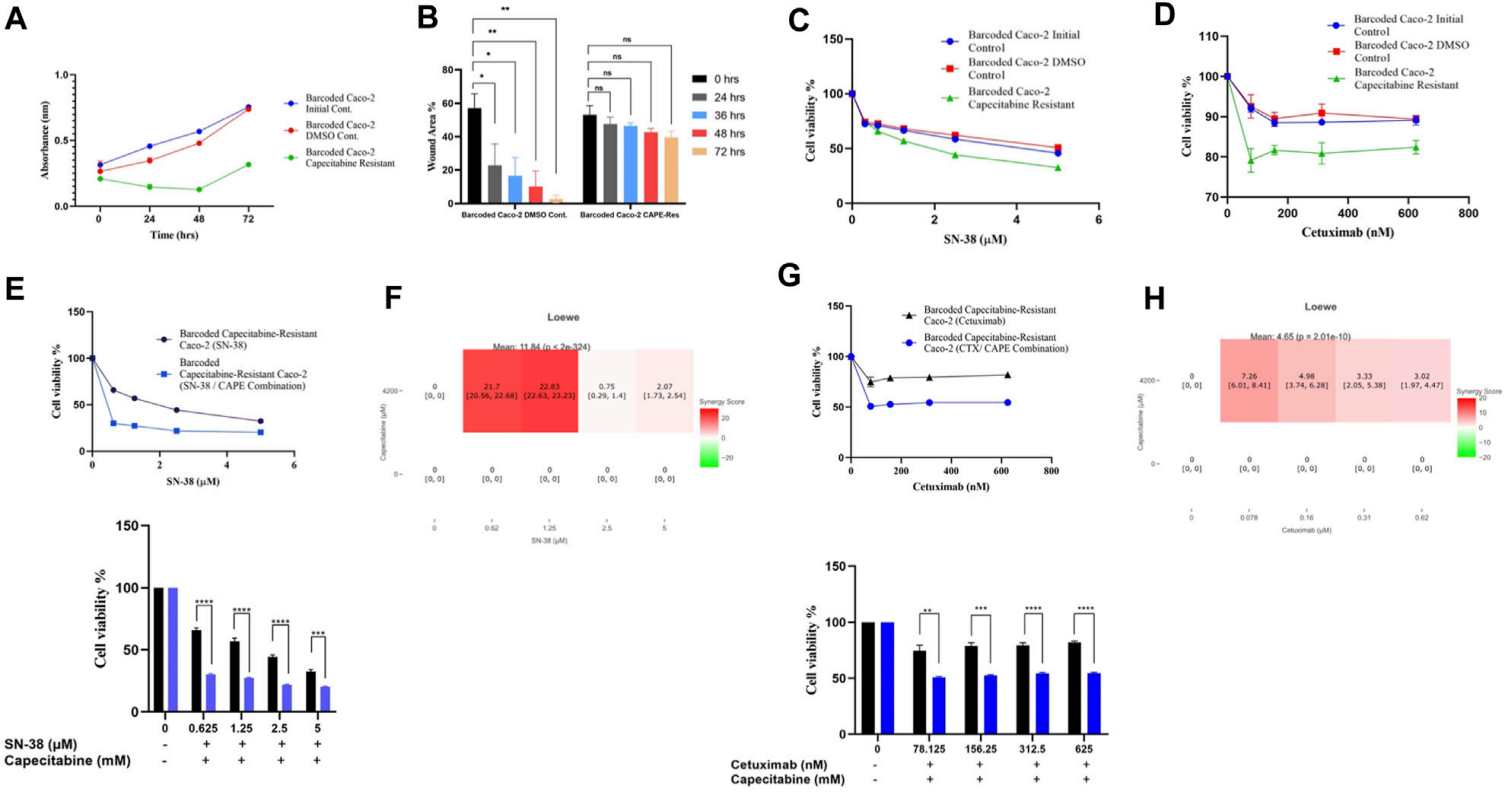
Collateral drug sensitivity was observed in capecitabine resistant Caco-2 cells. **(A)**. Growth rate measurements at 24 h intervals for capecitabine-resistant Caco-2 replicate B, DMSO, and initial cells. **(B)**. Scratch assay showing migration rates of capecitabine-resistant Caco-2 replicate B and DMSO control Caco-2 cell lines were measured based on the percentage of wound closure in 24, 36, 48, and 72 h intervals. **(C)**. Dose response curve analysis for capecitabine-resistant Caco-2 replicate B exhibited collateral sensitivity to SN-38. **(D)**. Dose response curve analysis in capecitabine-resistant Caco-2 replicate B showed collateral sensitivity to cetuximab. **(E)**. Combination of capecitabine and SN-38 in capecitabine-resistant Caco-2 replicate B demonstrated collateral sensitivity. **(F)**. Loewe score identified collateral sensitivity of capecitabine and SN-38 combination as an additive. **(G)**. Cetuximab and capecitabine combination exhibited collateral sensitivity in capecitabine-resistant Caco-2 replicate B. **(H)**. Synergy test performed using the Loewe score showed an additive effect of cetuximab and capecitabine combination for collateral sensitivity. Error bars represent SEM. *p < 0.5, **p < 0.01, ***p < 0.001 ****p < 0.0001.

The cost of drug resistance has been proposed as inducing sensitivity to second-line drugs, a phenomenon known as collateral drug sensitivity (Gatenby et al., 2009; Hughes and Andersson, 2015; Acar et al., 2020). The cost of adapting to capecitabine in Caco-2 cells may be reflected as an evolutionary trade-off in affecting growth rate in this scenario. We therefore reasoned to look at the difference in growth rate change in the capecitabine-resistant Caco-2 cell line replicate B, which had the highest resistance. Growth rate was found to be lower in barcoded capecitabine resistant Caco-2 cells compared to initial and control DMSO Caco-2 cell lines (Figure 4A). Furthermore, scratch assay to assess wound closure and hence migration abilities of cells demonstrated that capecitabine-resistant Caco-2 cells were slower when compared to DMSO control Caco-2 cells, indicating the effect capecitabine resistance in migratory abilities of drug resistant Caco-2 cells (Figure 4B). The next step was to look for potential collaterally sensitive drugs in the capecitabine-resistant Caco-2 cell line replicate B. To do so, we used irinotecan and S-38, two of the most commonly used chemotherapeutic drugs in treatment of CRC patients. We prioritized SN-38 testing because it is an active metabolite of irinotecan, and we hypothesized that SN-38 would be more effective at inducing collateral sensitivity. Collateral drug sensitivity testing using the MTT cell viability assay demonstrated increased sensitivity to SN-38 in Caco-2 cells when compared to control DMSO and initial Caco-2 cells (2.03- and 1.7-fold decrease in IC50 value, respectively) (Figure 4C). In the capecitabine-resistant Caco-2 cell line replicate B, irinotecan did not appear to induce collateral sensitivity, implying that pro-drug irinotecan may require additional time to be active in order to induce collateral drug sensitivity (Supplementary Figure S2A). Because Caco-2 cells have the KRAS WT genotype, we wanted to test another commonly used compound, the EGFR monoclonal antibody cetuximab, in KRAS WT CRC patients. As previously observed in SN-38, collateral drug sensitivity testing of cetuximab revealed a strong decrease in IC50 value in capecitabine-resistant Caco-2 B replicate compared to control DMSO and initial Caco-2 cell lines, implying that cetuximab induces collateral sensitivity as a potential second line drug in capecitabine-resistant KRAS WT cells (Figure 4D). These findings suggest that SN-38 and cetuximab are involved in inducing collateral sensitivity in capecitabine-resistant Caco-2 cells.

We then wondered if combining capecitabine with SN-38 and cetuximab would further increase collateral drug sensitivity. To investigate this effect, we first performed MTT cell viability assay in SN-38 alone and SN-38 in combination with capecitabine in capecitabine-resistant Caco-2 replicate B. This assay showed that combining SN-38 with capecitabine was more effective than SN-38 alone in inducing collateral sensitivity in capecitabine-resistant Caco-2 cells (4.3-fold decrease in IC50 values) (Figure 4E). To better understand the collateral sensitivity effect observed in this assay, we wanted to see if there was a synergy or an additive effect when drug combinations were used. For this purpose, we used the SynergyFinder v2.0 tool (University of Helsinki, Finland) and calculated synergy scores based on the Loewe score. According to the Loewe score, we observed a synergistic effect for the SN-38 and capecitabine combination at the lowest three concentrations (Figure 4F). Furthermore, the same approach was used for the capecitabine and cetuximab combination, where we observed improved collateral sensitivity in the presence of capecitabine and cetuximab combination in comparison to capecitabine alone (Figure 4G). Furthermore, it revealed the existence of an additive effect for cetuximab in inducing collateral sensitivity at all concentrations based on the Loewe score (Figure 4H). As a result, we were able to conclude that a collateral sensitivity induced by drug combinations and additive effects were present in inducing collateral sensitivity in SN-38 and cetuximab combination with capecitabine in capecitabine-resistant Caco-2 cells.

### Capecitabine and dabrafenib combination with irinotecan enhances collateral sensitivity in irinotecan resistant HT-29 cells

**FIGURE 5:**
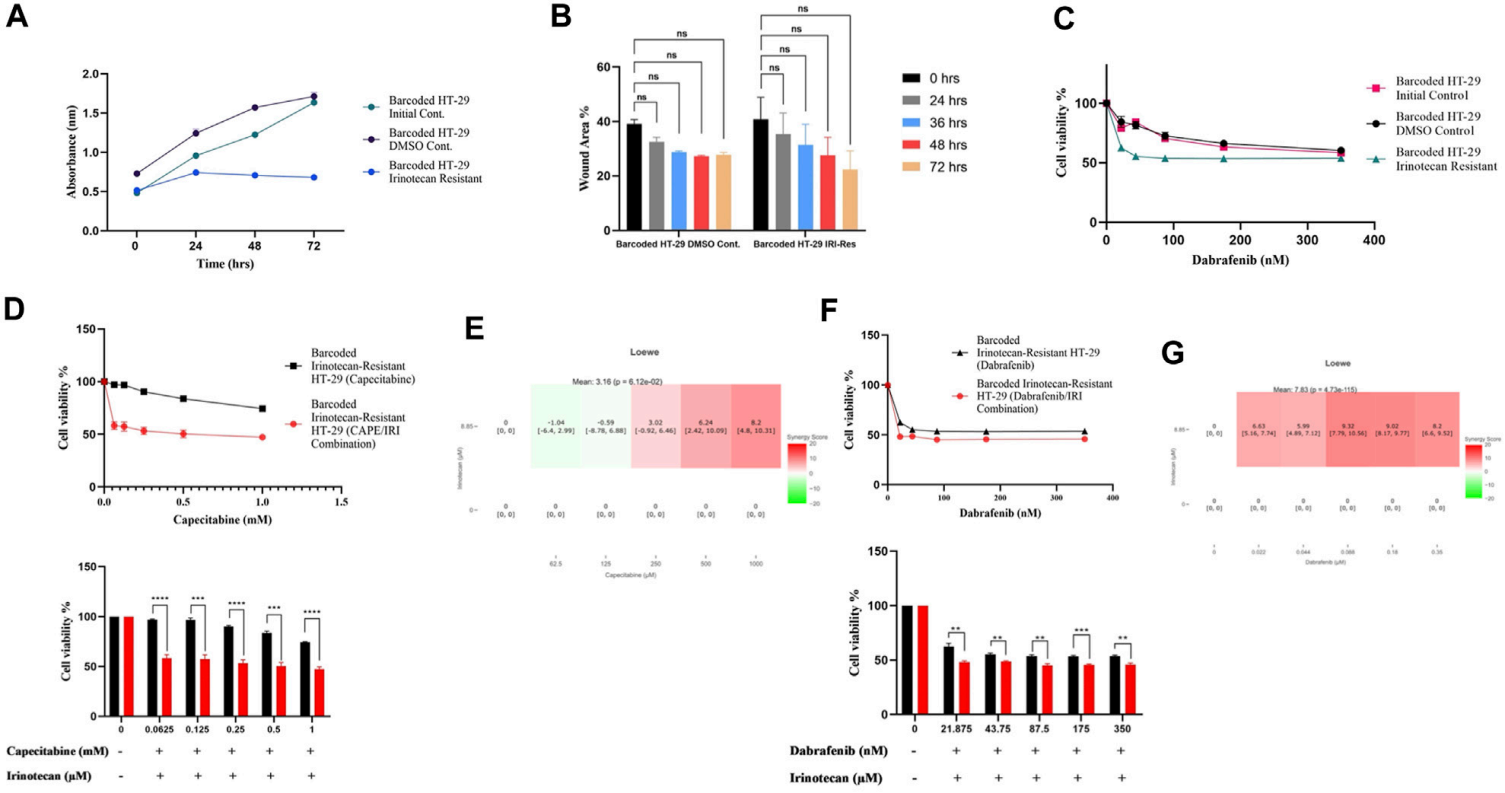
Collateral sensitivity was seen in irinotecan-resistant HT-29 cells. **(A)**. Measurement of growth rate differences at 24 h intervals for irinotecan-resistant HT-29 replicate B. **(B)**. Migratory abilities of irinotecan-resistant HT-29 replicate B and DMSO control HT-29 cell lines were measured based on the percentage of wound closure in 24h, 36h, 48h, and 72 h intervals. **(C)**. Collateral sensitivity to dabrafenib was identified in irinotecan-resistant HT-29 replicate B. **(D)**. Irinotecan and capecitabine combination exhibited collateral sensitivity in irinotecan-resistant HT-29 replicate B. **(E)**. Loewe score showed additive effect of irinotecan and capecitabine combination in inducing collateral sensitivity. **(F)**. Combination of irinotecan and dabrafenib demonstrated collateral sensitivity in irinotecan-resistant HT-29 replicate B. **(G)**. Synergy testing using Loewe score showed additive effects for irinotecan and dabrafenib combination in inducing collateral sensitivity in irinotecan-resistant HT-29 replicate B. Error bars represent SEM. *p < 0.5, **p < 0.01, ***p < 0.001 ****p < 0.0001.

As before, we wanted to see if irinotecan-induced resistance in HT-29 replicate B had a cost of resistance and thus collateral sensitivity to second-line drugs. We first wanted to check if irinotecan resistance affected growth rate as a cost of resistance in the most irinotecan resistant HT-29 replicate B. The growth rate assay revealed that HT-29 replicate B grew slower than the initial and control DMSO HT-29 cell lines (Figure 5A). Contrary to effect of capecitabine resistance in Caco-2 cells for slowing migration, irinotecan resistance did not exhibit a significant change in migration in HT-29 cells, presumably due to inefficiency in irinotecan to induce this phenotypic effect (Figure 5B). We hypothesized that the cost of resistance in these cells as seen in decreased growth and migration rates might induce collateral sensitivity to second line drugs. Capecitabine is a commonly used chemotherapeutic agent in the first-line treatment of CRC patients, so we wanted to see how it affected irinotecan-resistant HT-29 cells and if it could induce collateral sensitivity. Furthermore, HT-29 cell line with a V600E mutation in the BRAF gene has been shown to be sensitive to the BRAF V600E inhibitor dabrafenib. Although we expected HT-29 cells to be sensitive to dabrafenib, we wondered if dabrafenib could induce collateral sensitivity in irinotecan-resistant HT-29 cells. For this purpose, we first tested capecitabine’s collateral drug sensitivity on irinotecan-resistant replicate B, control DMSO, and initial HT-29 cell lines. The MTT cell viability results showed that capecitabine alone had no effect on inducing collateral sensitivity in irinotecan-resistant HT-29 cells when compared to control DMSO and initial HT-29 cell lines (Supplementary Figure S1B). When we applied the same approach to the effect of dabrafenib, we found that dabrafenib induced collateral sensitivity in irinotecan-resistant HT-29 cells when compared to control DMSO and initial HT-29 cell lines (Figure 5C). These results show that dabrafenib alone can provide collateral sensitivity in irinotecan-resistant HT-29 cells, whereas capecitabine alone as a second-line treatment is insufficient.

Next, we wanted to see if combining capecitabine and dabrafenib with irinotecan would facilitate or improve collateral sensitivity in HT-29 cells that were resistant to irinotecan. The MTT cell viability assay showed the effect of collateral sensitivity for combination of capecitabine, and irinotecan as indicated by 8.7-fold decrease in IC50 value, suggesting the importance of this drug combination for collateral sensitivity in irinotecan-resistant HT-29 cells (Figure 5D). Next, we asked whether the observed combination effects were due to synergy or additive effects by performing synergy score calculations. According to the Loewe score, the combination of capecitabine and irinotecan had an additive effect in all concentrations, as indicated by the SynergyFinder tool (Figure 5E). Furthermore, when we examined the effect of dabrafenib and irinotecan in the same group of cells, we observed that the combination effect was present but minor (Figure 5F). Last, according to the Loewe score, the combination of dabrafenib and irinotecan resulted in an additive effect (Figure 5G). As a result, capecitabine alone as second-line therapy was ineffective in providing collateral sensitivity in irinotecan-resistant HT-29 cells, whereas this effect was greatly enhanced by the combination of capecitabine and irinotecan, possibly due to the additive effect of these two compounds. Furthermore, dabrafenib alone appeared to be effective in inducing collateral sensitivity in irinotecan-resistant HT-29 cells; however, the combination of irinotecan and dabrafenib improved this effect only briefly, as it was supported by a minor additive effect at the lowest concentration of this combination.

## Discussion

In this study, we used cellular barcoding technique to measure drug resistance in the Caco-2 and HT-29 cell lines, which were resistant to capecitabine and irinotecan, respectively. Using this approach, we were able to identify polyclonal drug resistance that was caused by pre-existing and *de novo* barcodes, both from harvested end points where cells became resistant to capecitabine and irinotecan, and via temporal evolutionary dynamics from monthly medium in a non-destructive way. The predominance of evolving tumour populations in different tumour types, which evolve in a branching and linear pattern, as well as the evolution of tumour populations in response to therapy, support the polyclonal drug resistance observed in this study (Burrell and Swanton, 2014; Davis et al., 2017). Several studies have found evidence of polyclonal resistance involved in the selection of drug-resistant subclones especially in AML, ALL, CLL, glioma, and ovarian cancer as a plausible mechanism induced by chemotherapies (Burrell and Swanton, 2014). However, identifying true numbers of drug-resistant subclones has always been challenging due to the shortcomings of next-generation sequencing techniques and impure tumour populations and required better subclonal reconstruction approaches (Caravagna et al., 2020). For instance, barcoding technology improves the detection of haplotype frequencies 10^4^-fold, whereas next-generation sequencing methods can only detect haplotype frequencies at a precision of only 0.01%–0.1% (Acar et al., 2020). Hence, the barcoding method that we used in this study provided extensive understanding on the underlying capecitabine and irinotecan resistance mechanisms as well as the prevalence of tumour heterogeneity.

Understanding how drug resistance develops under the selection pressure of a drug needs thorough observation of the subclonal evolution landscape (Turajlic et al., 2019). An important aspect of the barcoding technology utilized in this study allowed us to track the temporal evolution of drug resistance using monthly collected media. This is often an overlooked problem in studying drug resistance mechanisms and is only utilised previously once *in vitro* (Acar et al., 2020); however broadly used in liquid biopsy studies of ctDNA or CTCs (Alix-Panabières and Pantel, 2021). Longitudinal clonal evolution presented in this study provided a power to monitor clonal dynamics of barcodes during the period of experimental plan. For example, when 1XIC50 concentration of capecitabine was applied to Caco-2 cells, we observed an increase in temporal barcode frequencies, but this was followed by a decrease in barcode frequencies in certain barcodes when the drug treatment was increased to 2XIC50 concentration of capecitabine. As a result, we were able to better understand the dynamic nature of drug resistance in an ongoing experimental setup using multiple data points rather than the conventional method of capturing it via a single end point.

Regardless of how effective monotherapy or, in some cases, combination therapy is, drug resistance is inevitable (Holohan et al., 2013). Combination therapy has modestly improved overall survival in cancer patients when compared to monotherapy as well as associated toxicity is one of the drawbacks of combination therapy (Mokhtari et al., 2017). Exploiting tumours’ vulnerabilities based on sequential drug treatment using the evolutionary standpoint, i.e., collateral drug sensitivity, has been shown to be promising recently in some studies, including ours (Zhao et al., 2016; Dhawan et al., 2017; Acar et al., 2020). As part of this study, the landscape of capecitabine and irinotecan pressure yielded actionable collateral sensitive drugs such as SN-38 and cetuximab for capecitabine resistant Caco-2 cells and dabrafenib for irinotecan resistant HT-29 cells. Intriguingly, we had to keep irinotecan present in HT-29 cells for capecitabine to provide collateral sensitivity. This could be explained by our observations of the additive effects of irinotecan and capecitabine, which we found using the SynergyFinder tool. Last, we wondered if multiple drug resistance might be one of the mechanism responsible for capecitabine and irinotecan mediated resistance in Caco-2 and HT-29 cell lines, respectively. To assess this possibility, we checked the mRNA expression levels of MDR-1 gene, namely, ABCB1 and found that ABCB1 mRNA expression was upregulated in capecitabine-resistant Caco-2 replicates A, B, C and irinotecan-resistant HT-29 replicates A, B, C in comparison to their DMSO control counterparts (Supplementary Figures S3, S4). Furthermore, our analysis to examine whether collaterally sensitive drug dabrafenib might affect barcode numbers in irinotecan-resistant HT-29 cell lines revealed a change in clonal dynamics of barcode distribution when irinotecan-resistant HT-29 cells were treated with IC50 (87.5 nM) concentration of dabrafenib, and barcode sequencing was performed (Supplementary Figure S5). Moreover, we found a loss in number of barcodes (number of lost barcodes; pre-existing: 2, 6, 2 in A, B, C; *de novo*: 26, 22, 22 in A, B, C, respectively) detected following 10 days of dabrafenib treatment (Supplementary Table S5).

Despite the improved resolution that we aimed to achieve by using barcoding technology to monitor drug resistance in our system, we recognize its limitations and therefore potential improvements. First, the system we studied was based solely on 2 cell lines and lacked tumour microenvironment components such as cancer-associated fibroblasts (CAFs) and immune cells. Given the important roles of CAFs in mitigating drug sensitivity and immune cells in immune evasion (Li et al., 2015; Khalaf et al., 2021), identifying clonal dynamics with the presence of these stromal cells and measuring barcode frequency changes would be intriguing. Second, the detected barcodes in our study were unable to demonstrate phenotypes associated with drug resistance, suggesting that additional work is needed that establishment of single barcode derived drug resistant cell lines may vary in term of their collateral drug sensitivities. Third, the population size and drug concentrations used in this study may not accurately reflect what is observed and administered in the clinic, indicating the need for technological advancements *in vitro* setting that may better reflect the disease. Fourth, collateral sensitivity testing was originally studied to understand antibiotic resistance in bacteria included much more parallel experimental replicates than the three used in this study (Nichol et al., 2015), and hence a similar approach will be needed to better identify or rule out stochastic changes in the evolution of drug resistance in cancer. Fifth, validation of our findings will be needed, particularly in highly patient-relevant model systems such as patient-derived organoids (Vlachogiannis et al., 2018), which can also incorporate cellular barcoding to determine the drug response observed in organoids alongside high resolution barcode frequency measurements. Despite its limitations, the model system presented in this study will allow researchers to approach the drug resistance problem by incorporating cellular barcoding technology and considering evolutionary therapy with the ultimate goal of identifying second-line therapeutics that can sensitize drug-resistant populations.

## Data Availability

The datasets presented in this study can be found in online repositories. The names of the repository/repositories and accession number(s) can be found below: https://www.ebi.ac.uk/; E-MTAB-12818.

## Supplementary material

Supplementary Figure 1 Barcode frequency measurements for sensitive barcodes. **(A)**. Detected sensitive barcode frequencies for capecitabine-resistant Caco-2 cell line replicate B was shown. **(B)**. Sensitive barcode frequencies in irinotecan-resistant HT-29 cell line replicate B was demonstrated.

Supplementary Figure 2 No collateral sensitivity was observed for irinotecan and capecitabine. **(A)**. Dose response curve analysis for identification of collateral sensitivity to irinotecan in capecitabine-resistant Caco-2 cell line replicate B, and **(B)**. to capecitabine in irinotecan-resistant HT-29 cell line replicate B did not exhibit a collateral sensitivity.

Supplementary Figure 3 mRNA expression analysis of ABCB1 in capecitabine-resistant Caco-2 cells and DMSO control. ABCB1 gene expression was upregulated in capecitabine-resistant Caco-2 replicates A, B and C relative to DMSO control.

Supplementary Figure 4 mRNA expression analysis of ABCB1 in irinotecan-resistant HT-29 cells and DMSO control. ABCB1 gene expression was upregulated in irinotecan-resistant HT-29 replicates A, B and C relative to DMSO control.

Supplementary Figure 5 Barcode frequency measurements in irinotecan-resistant HT-29 cells after dabrafenib treatment. Barcode frequency calculations were carried out in irinotecan-resistant HT-29 cell line replicates A, B and C following treatment with IC50 (87.5 nM) concentration of dabrafenib for 10 days.

Supplementary Table 1 Resistance fold change calculations in capecitabine-resistant Caco-2 cell lines replicates A, B, and C in comparison to DMSO control Caco-2 cell line were shown.

Supplementary Table 2 Fold changes in resistance in irinotecan-resistant HT-29 cell line replicates A, B, and C relative to DMSO control HT-29 cell line were demonstrated.

Supplementary Table 3 Pre-existing and *de novo* resistant barcode numbers in capecitabine-resistant Caco-2 cell line replicates A, B, and C.

Supplementary Table 4 Pre-existing and *de novo* resistant barcode numbers in irinotecan-resistant HT-29 cell line replicates A, B, and C.

Supplementary Table 5 Barcode numbers in irinotecan-resistant HT-29 cell line replicates A, B, and C following dabrafenib treatment for 10 days.
