# Supplementary figures and images for: Identification of collateral sensitivity and evolutionary landscape of chemotherapy-induced drug resistance using cellular barcoding technology
Source: Front Pharmacol. 2023 Jul 11;14:1178489. doi: 10.3389/fphar.2023.1178489 (PMC10366361; doi:10.3389/fphar.2023.1178489)

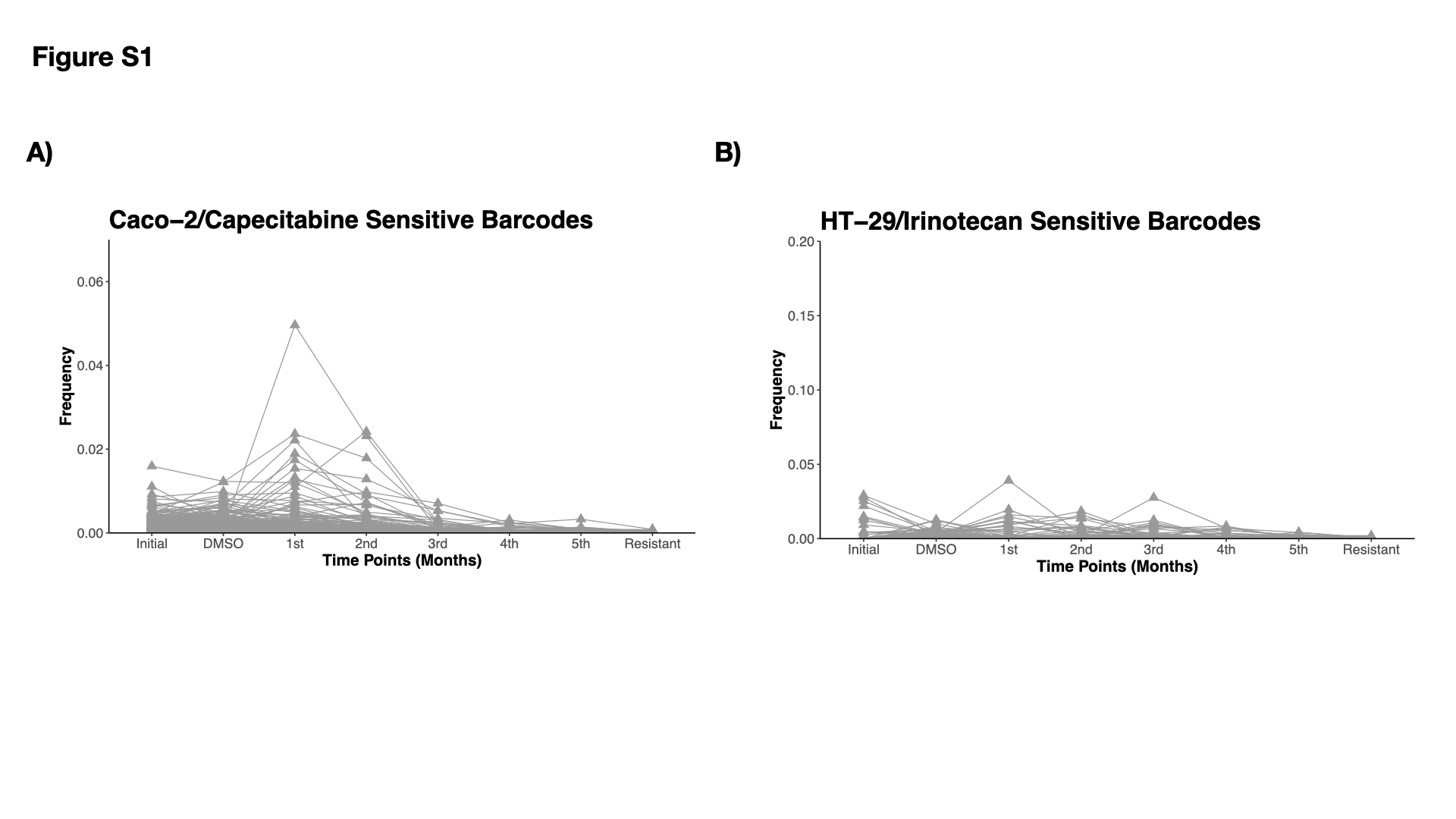

Supplement: Supplementary file 1 [file DataSheet1.zip › Figure S1.JPEG]

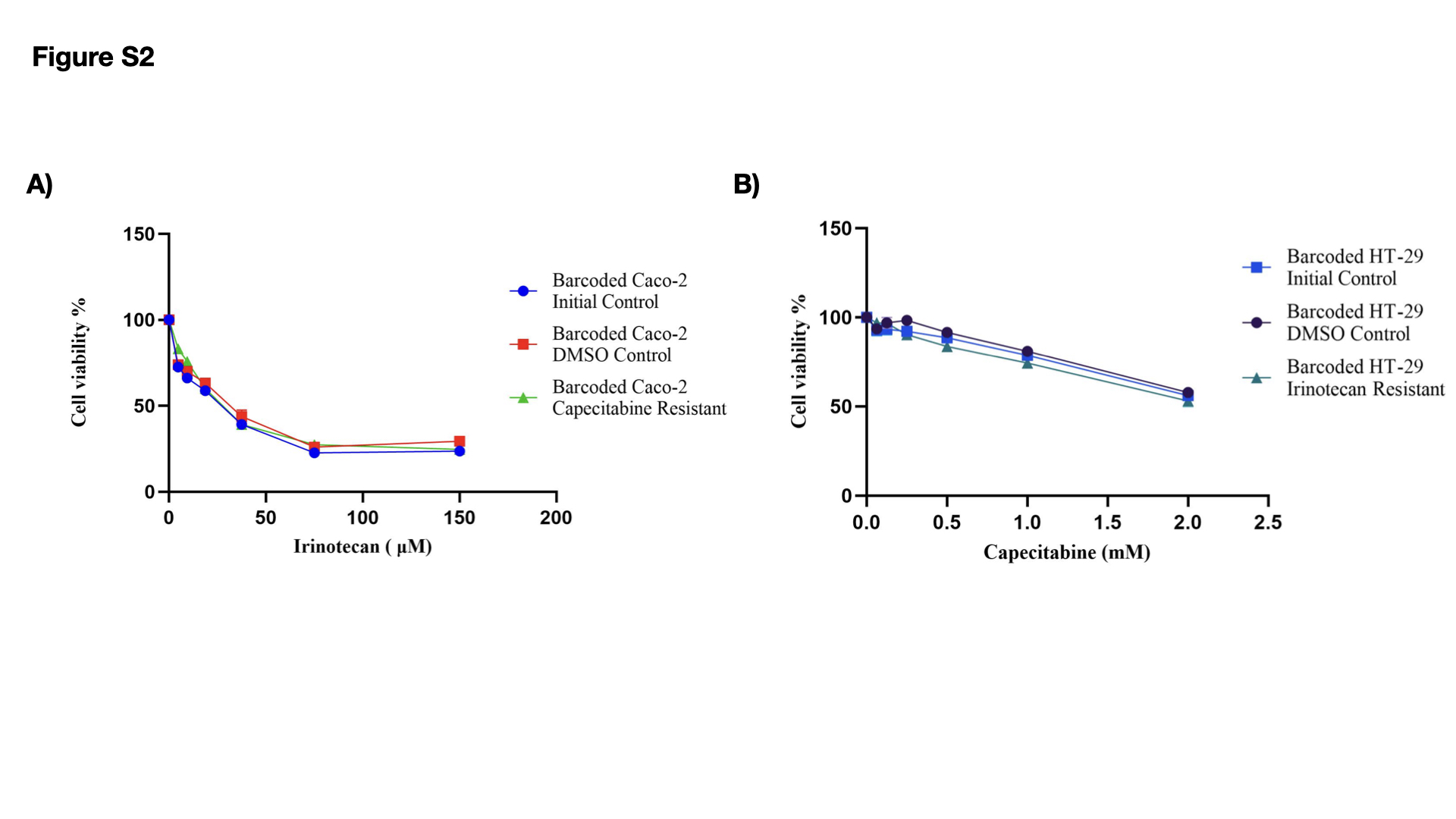

Supplement: Supplementary file 1 [file DataSheet1.zip › Figure S2.JPEG]

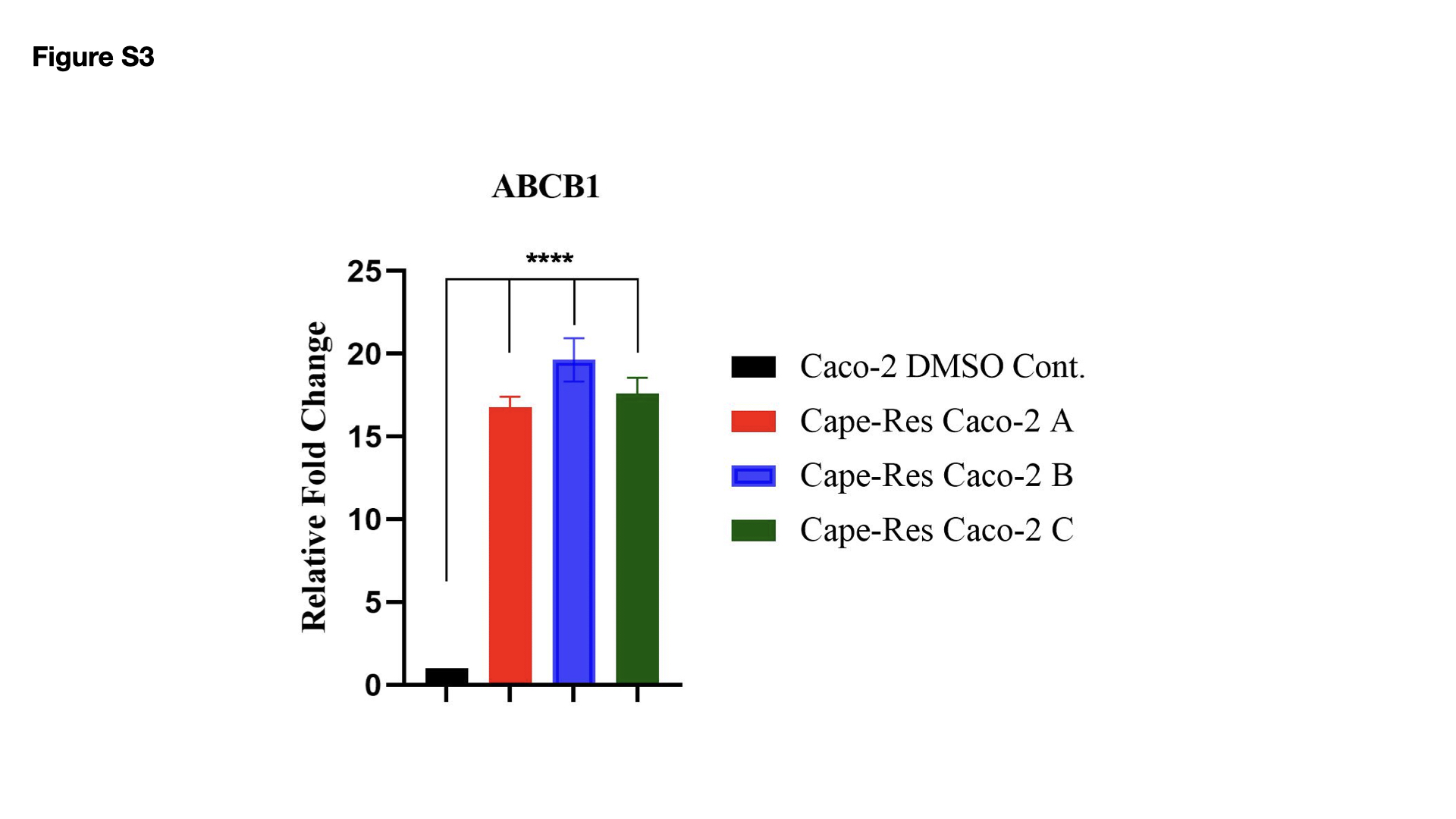

Supplement: Supplementary file 1 [file DataSheet1.zip › Figure S3.JPEG]

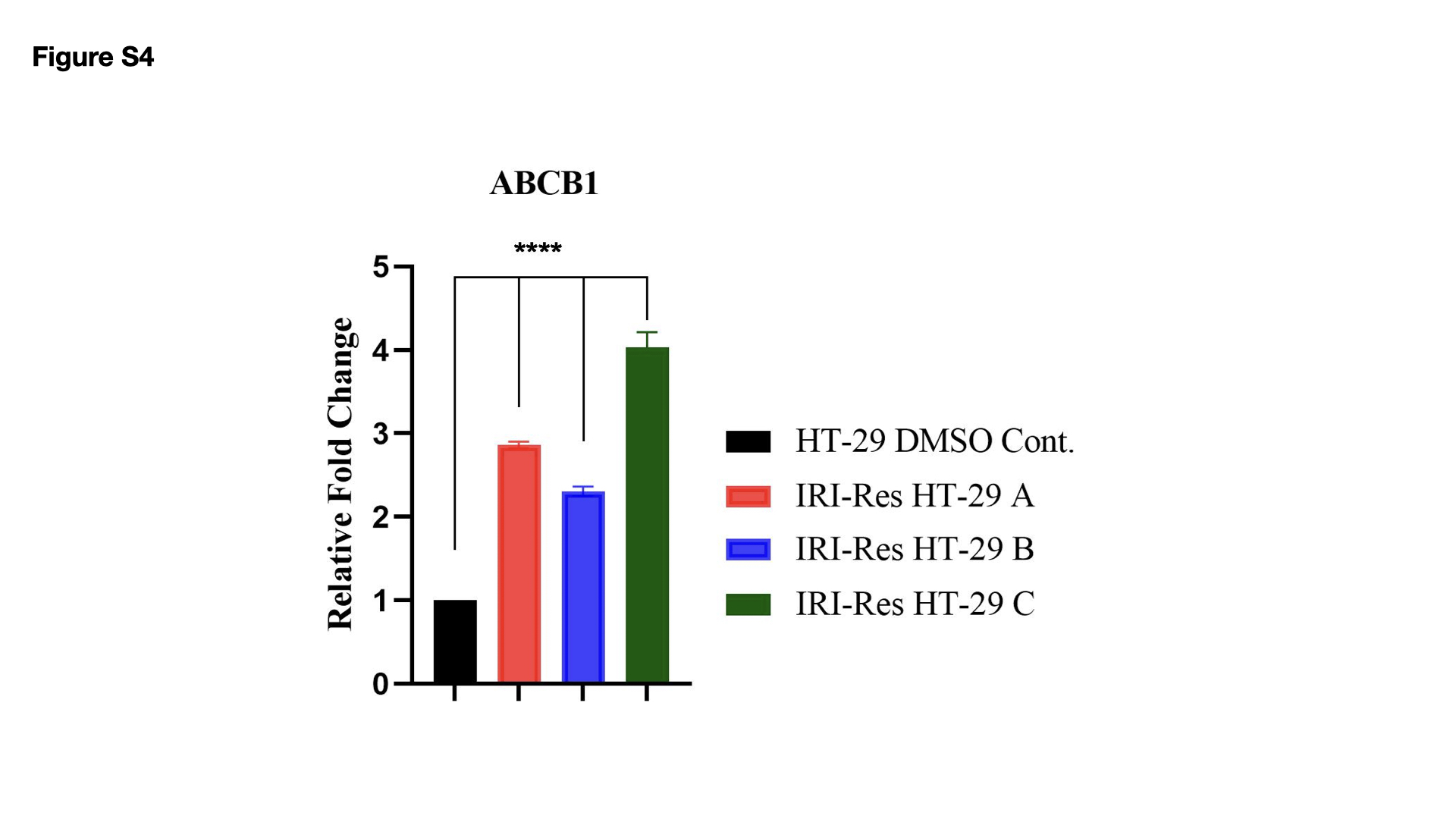

Supplement: Supplementary file 1 [file DataSheet1.zip › Figure S4.JPEG]

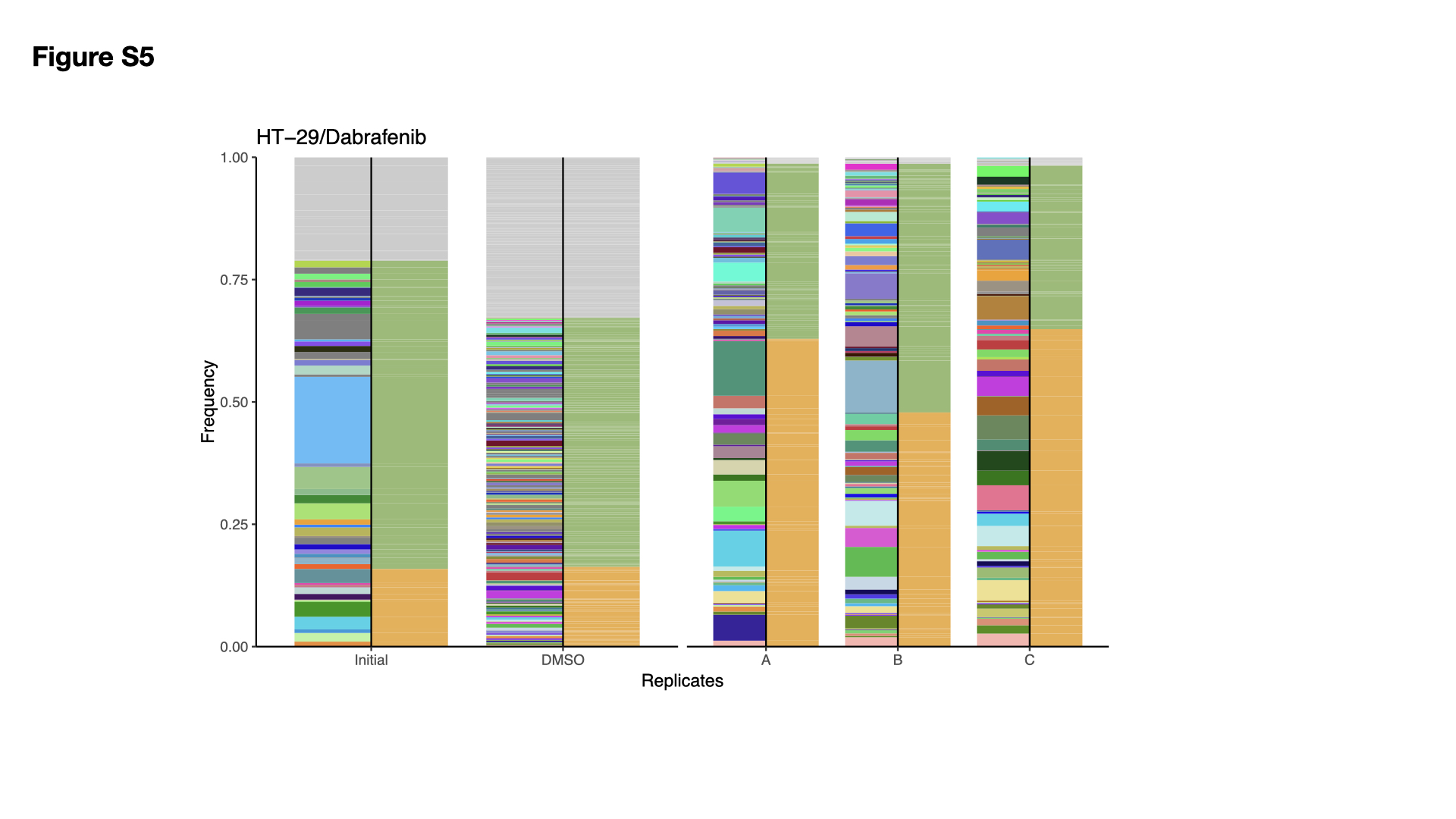

Supplement: Supplementary file 1 [file DataSheet1.zip › Figure S5.JPEG]

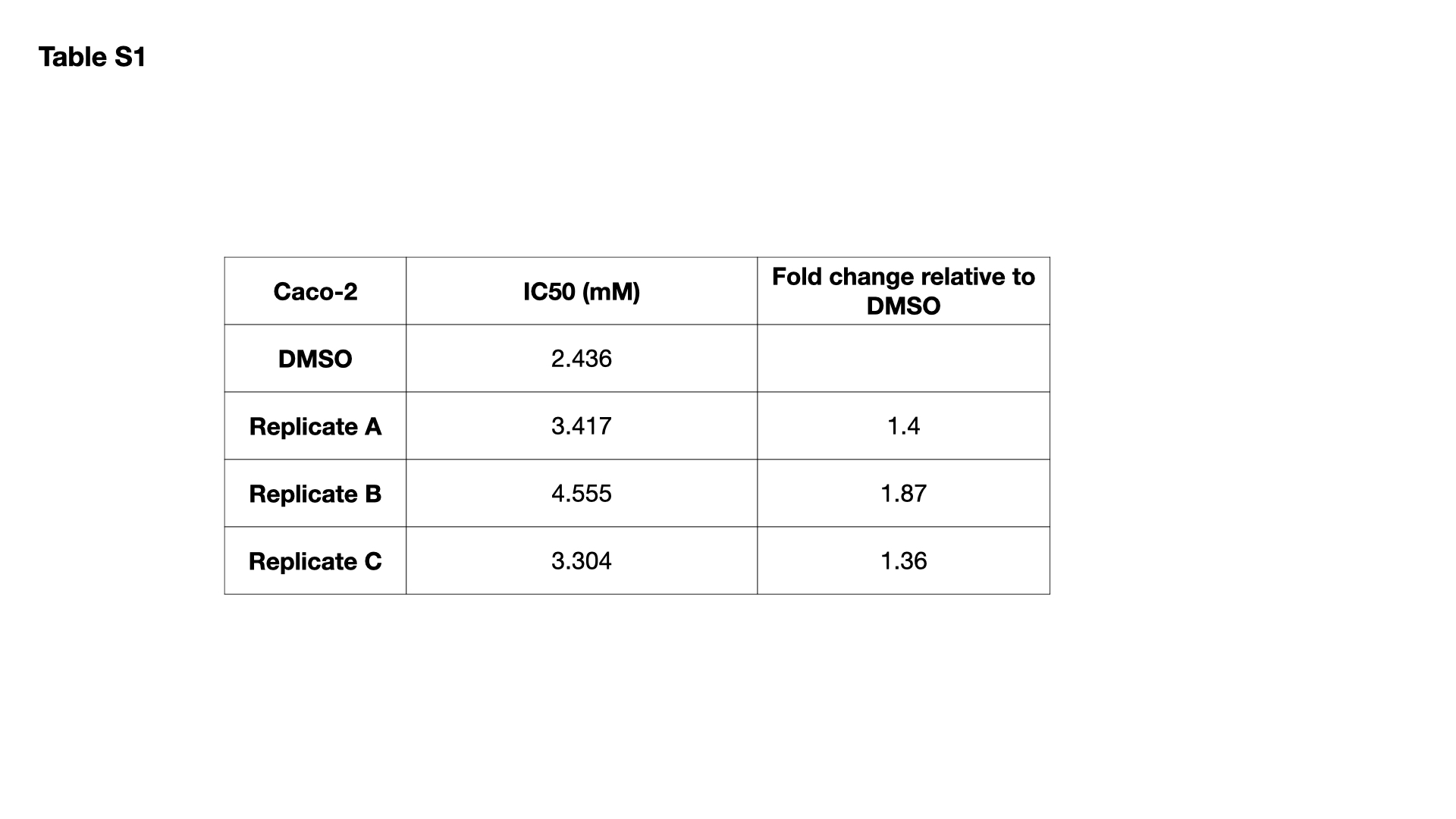

Supplement: Supplementary file 2 [file DataSheet2.zip › Table S1.JPEG]

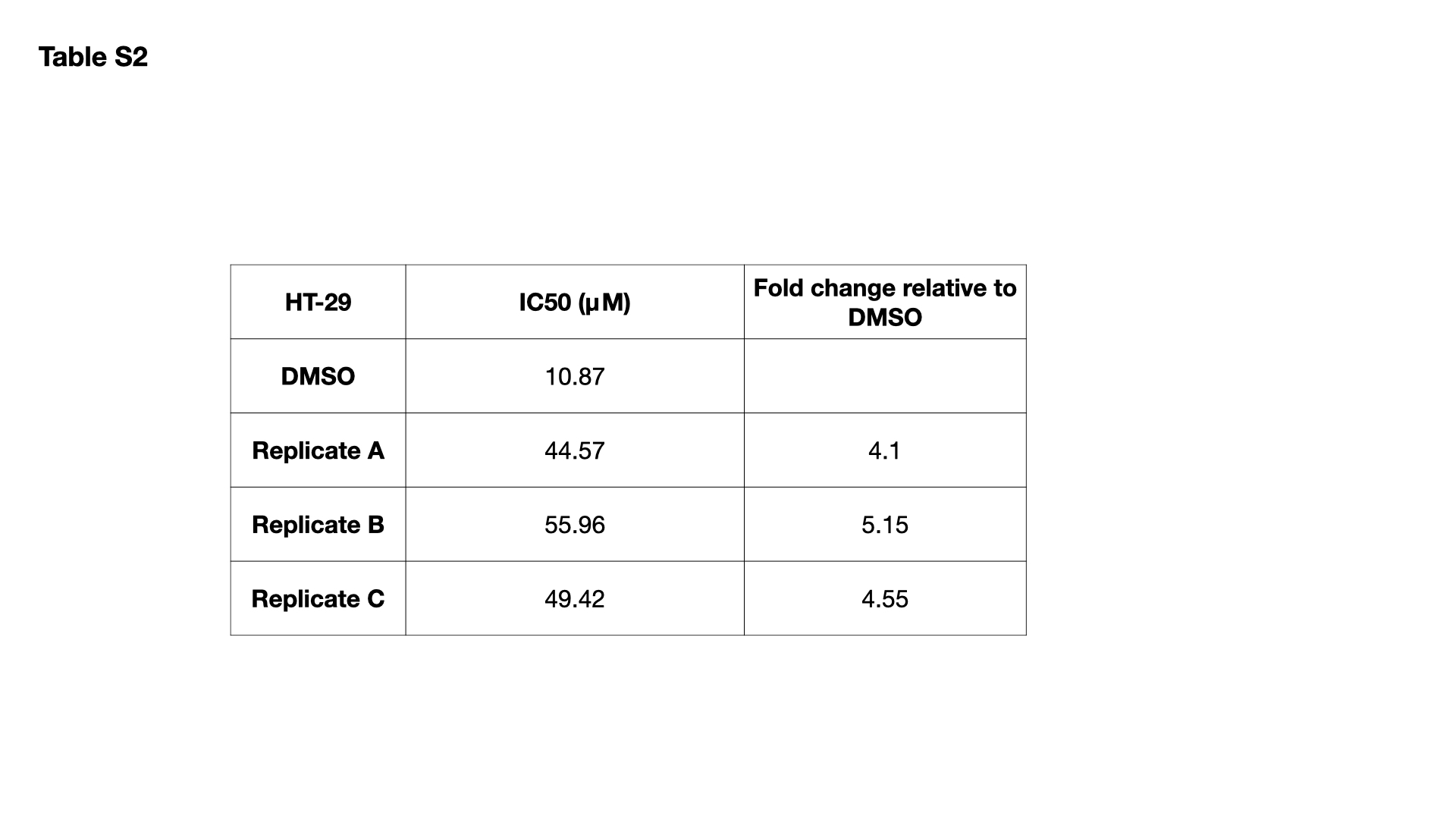

Supplement: Supplementary file 2 [file DataSheet2.zip › Table S2.JPEG]

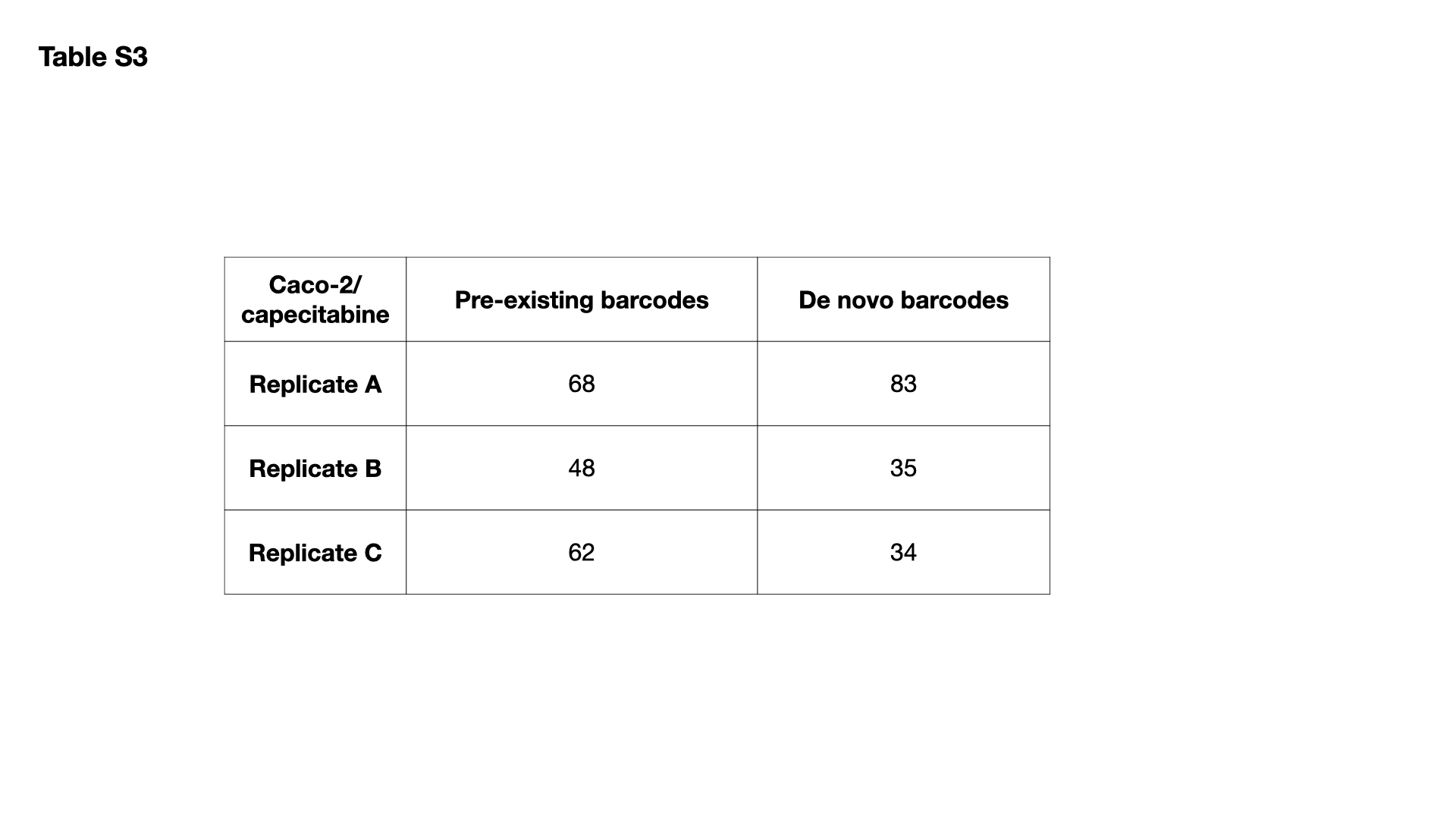

Supplement: Supplementary file 2 [file DataSheet2.zip › Table S3.JPEG]

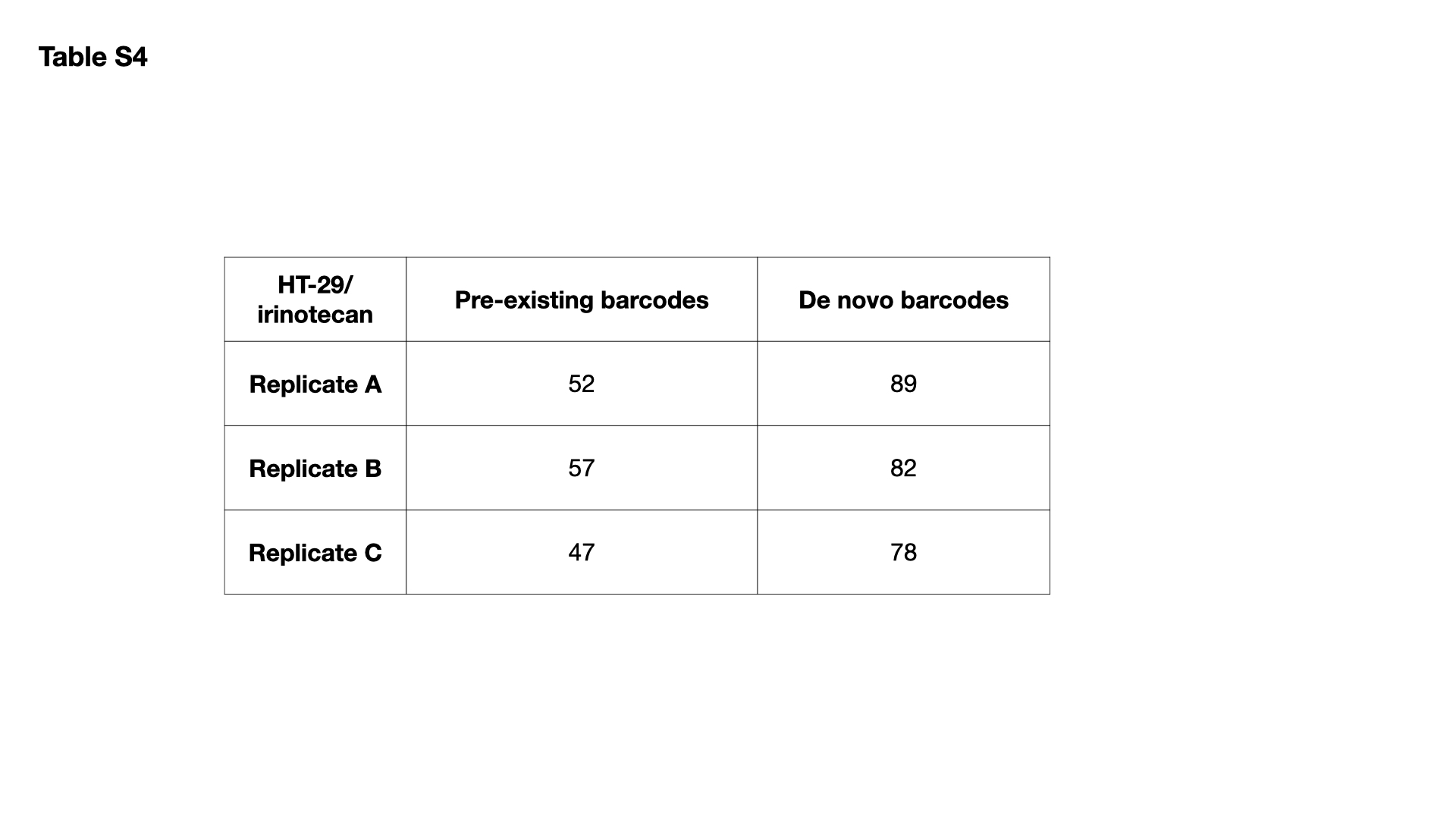

Supplement: Supplementary file 2 [file DataSheet2.zip › Table S4.JPEG]

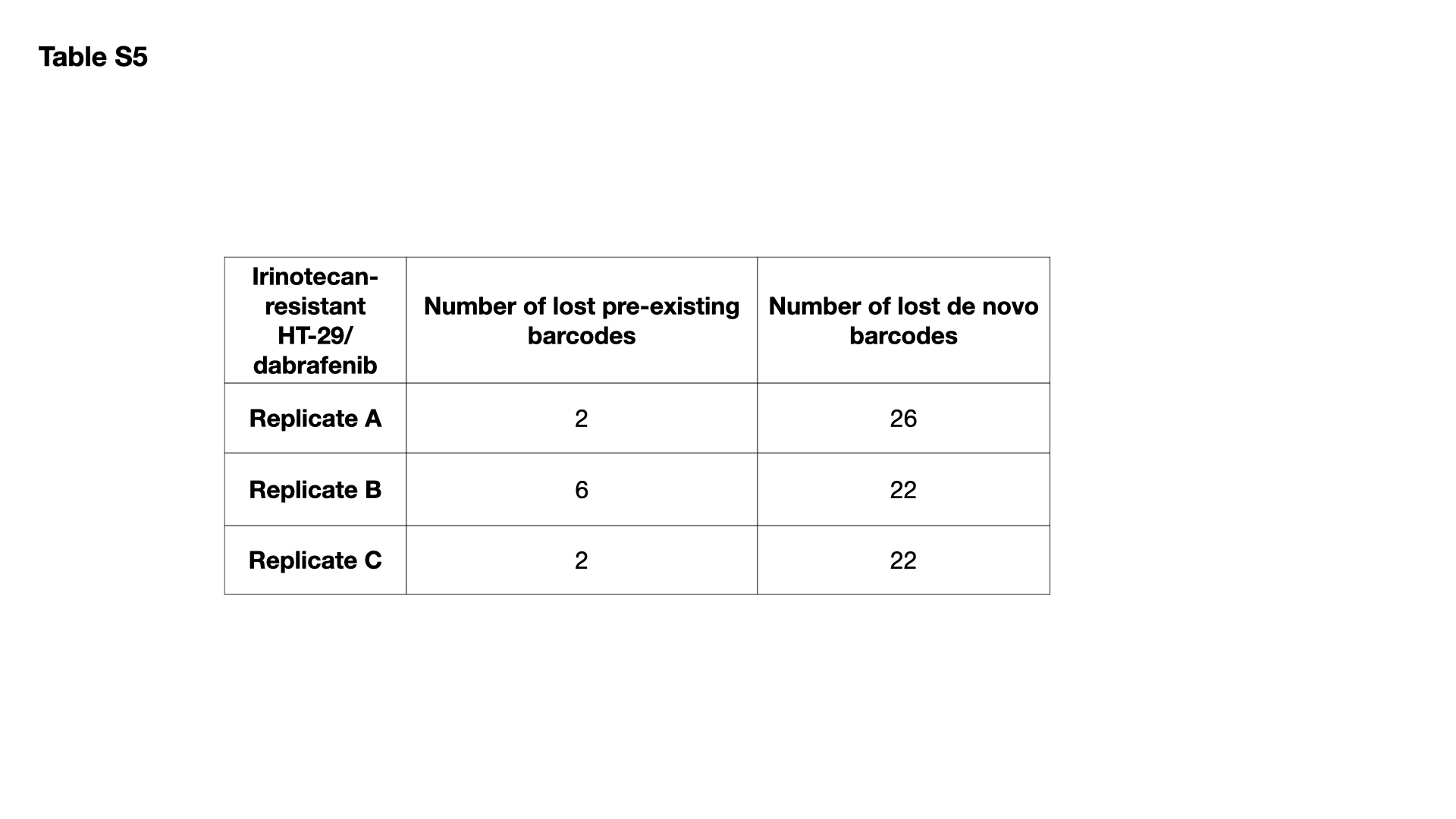

Supplement: Supplementary file 2 [file DataSheet2.zip › Table S5.JPEG]
